# A hybrid landmark Aalen-Johansen estimator for transition probabilities in partially non-Markov multi-state models

**DOI:** 10.1007/s10985-021-09534-4

**Published:** 2021-09-30

**Authors:** Niklas Maltzahn, Rune Hoff, Odd O. Aalen, Ingrid S. Mehlum, Hein Putter, Jon Michael Gran

**Affiliations:** 1 Oslo Centre for Biostatistics and Epidemiology, Oslo University Hospital, Oslo, Norway; 2 Oslo Centre for Biostatistics and Epidemiology, Department of Biostatistics, University of Oslo, Oslo, Norway; 3 National Institute of Occupational Health, Oslo, Norway; 4 Leiden University Medical Center, Leiden University, Leiden, The Netherlands

**Keywords:** Landmarking, Non-Markov multi-state models, Sick leave, The Aalen-Johansen estimator, Transition probabilities

## Abstract

Multi-state models are increasingly being used to model complex epidemiological and clinical outcomes over time. It is common to assume that the models are Markov, but the assumption can often be unrealistic. The Markov assumption is seldomly checked and violations can lead to biased estimation of many parameters of interest. This is a well known problem for the standard Aalen-Johansen estimator of transition probabilities and several alternative estimators, not relying on the Markov assumption, have been suggested. A particularly simple approach known as landmarking have resulted in the Landmark-Aalen-Johansen estimator. Since landmarking is a stratification method a disadvantage of landmarking is data reduction, leading to a loss of power. This is problematic for “less traveled” transitions, and undesirable when such transitions indeed exhibit Markov behaviour. Introducing the concept of partially non-Markov multi-state models, we suggest a hybrid landmark Aalen-Johansen estimator for transition probabilities. We also show how non-Markov transitions can be identified using a testing procedure. The proposed estimator is a compromise between regular Aalen-Johansen and landmark estimation, using transition specific landmarking, and can drastically improve statistical power. We show that the proposed estimator is consistent, but that the traditional variance estimator can underestimate the variance of both the hybrid and landmark estimator. Bootstrapping is therefore recommended. The methods are compared in a simulation study and in a real data application using registry data to model individual transitions for a birth cohort of 184 951 Norwegian men between states of sick leave, disability, education, work and unemployment.

**Supplementary Information:**

The online version contains supplementary material available at 10.1007/s10985-021-09534-4.

## Introduction

Multi-state models are increasingly being used to model complex epidemiological and clinical outcomes over time. One example is in the analysis of long-term sick leave and health related absence from work, where detailed longitudinal data on individuals are available through administrative registries (see e.g. Hoff et al. 2018). Multi-state models extend traditional hazard based time-to-event models to situations with a higher, finite, number of states, each of which defines a possible competing risks situation (Hougaard 1999; Andersen and Keiding 2002; Putter et al. 2007; Meira-Machado et al. 2008). In such models, the main parameters of interest are typically not the transition hazards, but rather the overall occupation and transition probabilities between states over time. In a Markov multi-state model, occupation and transition probabilities can be estimated consistently as a plug-in estimate based on the estimated transition intensities using the Aalen-Johansen (AJ) estimator (see Aalen et al. (2008)). However, for non-Markov models, the AJ estimator is only consistent for occupation probabilities (Datta and Satten 2001; Glidden 2002; Overgaard 2019; Nießl et al. 2020).

Several methods have been proposed for estimating transition probabilities in general semi- and non-Markov multi-state models based on subsampling (de Uña-Álvarez and Meira-Machado 2015; Allignol et al. 2014; Titman 2015; Putter and Spitoni 2018). They differ in that they are valid for models of different level of complexity. For example, the models of Titman (2015) and Putter and Spitoni (2018) are valid for general multi-state models, while the model of de Uña-Álvarez and Meira-Machado (2015) can be used for progressive multi-state models. The landmark Aalen-Johansen (LMAJ) method of Putter and Spitoni (2018) is based on analysing a subset of the population being in a specific state at a specific time point. This reference time and state is referred to as a landmark. Applying the AJ estimator to this landmark subset gives consistent estimates of transition probabilities from the landmark state at the landmark time, also for non-Markov models. A consequence of stratification to a landmark sample is that these subsets may become small, leading to increased variance and possibly unreliable point estimates. In this paper we suggest an alternative approach, the hybrid landmark Aalen-Johansen (HAJ) estimator, for models consisting of Markov and non-Markov transitions. We show that the proposed estimator is consistent, but that the traditional variance estimator can underestimate the variance. We therefore recommend using bootstrapping. The proposed hybrid estimator is based on a transition wise consideration of whether to use data from the landmark subsample or all available data in the estimation procedure. Inspired by Titman and Putter (2020) a type of two-sample test is suggested to select which transitions that are Markov (or close to Markov) and which are not. The resulting HAJ estimator can be seen as a compromise between the two extremes of either assuming all transitions are Markov or no transitions are Markov. As our results demonstrate, the HAJ estimator will, typically, have less bias than the AJ estimator and higher precision than the LMAJ estimator.

The outline for the paper is as follows. In Sect. 2 we define partially non-Markov multi-state processes and present the HAJ estimator. In Sect. 3 we give a heuristic justification of the estimator, discuss large sample properties and how tests of Markov behaviour following Titman and Putter (2020) can be used to construct the HAJ estimator. In Sect. 4 we consider a simulation study comparing the HAJ estimator to the AJ and LMAJ estimators. In Sect. 5 we apply the techniques to data from a Norwegian birth cohort to model sickness absence and work participation over time. A discussion is found in Sect. 6. R code for implementation and reproduction of the simulation study is available on GitHub (see Supporting Information).

## A hybrid landmark Aalen-Johansen estimator

**Fig. 1:**
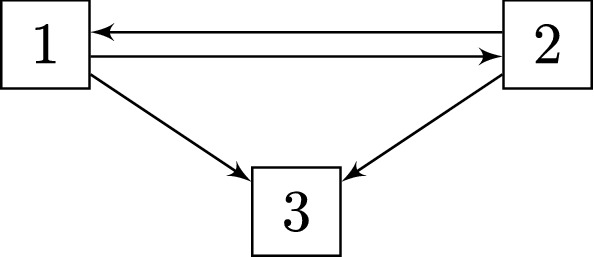
An illness-death model with recovery, where, for example, state 1 corresponds to employment, state 2 to sick leave and state 3 to permanent disability

Let us consider a multi-state model *X*(*t*) over a bounded time interval $[0, \tau ]$, taking values in the state space $\mathcal {K} = \{1, \ldots , K\}$. Let $E \subset \mathcal {K} \times \mathcal {K}$ be the set of possible transitions of *X*. An example of such a multi-state model, *X*(*t*), is the illness-death model with recovery, as illustrated in Fig. 1. This model consists of three states, say employment, sick leave and permanent disability, and four possible transitions between them. Note, however, that what follows hold for any multi-state model with a finite state space.

Say that for a given state $l \in \mathcal {K}$, we are interested in the transition probabilities from *l* to each of the states in $\mathcal {K}$, given by (for $s \le t$)

1 $$\begin{aligned} \mathbf {P}_l(s, t) := \left( P_{l1}(s,t). \ldots , P_{lK}(s,t) \right) ^\top , \end{aligned}$$

where $P_{lk}(s,t) := \mathsf {P}(X(t) = k \, | \,X(s) = l)$ and $k \in \{1,\ldots ,K\}$. For example, for the model in Fig. 1, $P_{21}(s,t)$ is the probability of being in work at time *t*, given sick leave at time *s*. The problem of interest is now to produce a consistent estimator of (1). For the sake of simplicity we consider the case without censoring. Censoring will briefly be discussed in section 3.3.

Note that the theory that follows is valid also for more than one landmark state *l*, so that *l* in practice can be a set of states. However, to ease notation, we focus on the most common scenario where *l* is one particular state in the state space $\mathcal {K}$.

### The Aalen-Johansen estimator

When the multi-state process is Markov, a consistent estimator of these transition probabilities is provided by the Aalen-Johansen estimator (see Aalen and Johansen 1978). For this, consider *n* i.i.d. realisations $X_i(t)$ of *X*(*t*), where for subject *i* we define the at risk process for state *j* as $Y_j^{(i)}(t) = 1\{ X_i(t-) = j\}$ and the transition counting process for transition $j \rightarrow k \in E$ as $N_{jk}^{(i)}(s,t) = \sum _{u \in (s,t]} 1\{X_i(u-) = j, X_i(u) = k\}$. We assume that all transition counting processes are locally finite i.e. at most a finite number of transitions happens on bounded intervals, hence justifying the summation index for the counting process just defined. Then, the aggregated at risk process

$$\begin{aligned}&\overline{Y}_{j}(t) := \sum _{i = 1}^{n} 1\{X_i(t-) = j\} \end{aligned}$$

corresponds to the number of individuals in state *j* just before time *t*, and the transition counting processes

$$\begin{aligned}&\overline{N}_{jk}(t) := \sum _{i = 1}^{n} \sum _{u \in (s,t]} 1\{X_i(u-) = j, X_i(u) = k\}, \end{aligned}$$

corresponds to the number of transitions directly from state *j* to state *k* in the interval (*s*, *t*]. Let $\overline{\mathbf {Y}}(t) := (\overline{Y}_{1}(t), \ldots , \overline{Y}_{K}(t))$ and $\overline{Y}_{\bullet }(t) = \sum _{j=1}^K \overline{Y}_j(t)$ be the total number of subjects at risk at time *t*. For $J_{j}(t) := 1\{\overline{Y}_j(t) > 0\}$ let

2 $$\begin{aligned} \widehat{{\varLambda }}_{jk}(t) := \int _{s}^{t} \frac{J_{j}(u) \mathrm {d}\overline{N}_{jk}(u)}{\overline{Y}_{j}(u)} \end{aligned}$$

be the Nelson-Aalen estimator of the transition rates and $\widehat{{\varvec{{\Lambda }}}}(t)$ a $K \times K$ matrix with (*j*, *k*)th element ($j \ne k$) equal to $\widehat{{\varLambda }}_{jk}(t)$ and diagonal elements $\widehat{{\varLambda }}_{jj}(t) = - \sum _{k \not = j} \widehat{{\varLambda }}_{jk}(t)$. The Aalen-Johansen (AJ) estimator of the transition probability matrix $\mathbf {P}(s, t)$, with elements $P_{jk}(s,t)$, is then given by

$$\begin{aligned} \widehat{\mathbf {P}}^{\text {AJ}}(s,t)&:= \prod _{u \in (s,t]} \left( \mathbf {I} + \varDelta \widehat{{\varvec{{\Lambda }}}}(u) \right) \end{aligned}$$

where the product is ordered according to the time ordering and consist of a finite number of terms due to the locally finite behaviour of the counting processes. Estimated state occupation probabilities at time *t* may be obtained by

3 $$\begin{aligned} \widehat{{\pi }}^{\text {AJ}}(t) = \widehat{{\pi }}(0) \widehat{\mathbf {P}}^{\text {AJ}}(0,t), \end{aligned}$$

where $\widehat{{\pi }}(0)$ is the row vector of empirical state occupation probabilities at $t=0$, given by $\widehat{\pi }_j(0) := \overline{Y}_j(0+) / \overline{Y}_{\bullet }(0+)$. Here, the $+$ in $(t+)$ means that the numbers observed to be in state *j* *at* time *t*, rather than just before time *t* are to be taken. Datta and Satten (2001) argued that the estimated state occupation probabilities in (3) are consistent, even if the multi-state model is non-Markov. The Aalen-Johansen (AJ) estimator of the transition probabilities $\mathbf {P}_l(s, t)$ from (1) is then given by

$$\begin{aligned} \widehat{\mathbf {P}}_{l}^{\text {AJ}}(s,t)&:= e_{l} \prod _{u \in (s,t]} \left( \mathbf {I} + \varDelta \widehat{{\varvec{{\Lambda }}}}(u) \right) , \end{aligned}$$

where $e_{l}$ is a vector with the *l*th element equal to 1, and all other elements 0.

### The landmark Aalen-Johansen estimator

Building on the results of Datta and Satten (2001), Putter and Spitoni (2018) defined the landmark Aalen-Johansen (LMAJ) estimator of transition probabilities. This estimator uses the landmark population $\{i: X_i(s) = l\}$ for estimation of the transition intensities. In what follows we consider the landmark time *s* and landmark state *l*, on which we condition, as fixed. Suppressing the dependence on *s* and *l* in the notation, we defining the landmark counting process and at risk process as $Y_j^{(i, \text {LM})}(t) := 1\{X_i(s) = l\}Y_j^{(i)}(t)$ and $N_{jk}^{(i, \text {LM})}(t) := 1\{X_i(s) = l\}N_{jk}^{(i)}(s,t)$. We define the aggregated at risk and counting processes based on the landmark population as

$$\begin{aligned} \overline{Y}_{j}^{(\text {LM})}(t) := \sum _{i = 1}^{n} Y_j^{(i, \text {LM})}(t) \quad \text {and} \quad \overline{N}_{jk}^{(\text {LM})}(t) := \sum _{i = 1}^{n} N_{jk}^{(i, \text {LM})}(s,t). \end{aligned}$$

Let $$\overline{\mathbf {Y}}^{(\text {LM})}(t) := (\overline{Y}_{1}^{(\text {LM})}(t), \ldots , \overline{Y}_{K}^{(\text {LM})}(t))$$ and $$\overline{Y}_{\bullet }^{(\text {LM})}(t) := \sum _{j = 1}^{K} \overline{Y}_{j}^{(\text {LM})}(t)$$.

For $J_{j}^{(\text {LM})}(t) := 1\{\overline{Y}_{j}^{(\text {LM})}(t) > 0\}$ define the landmark Nelson-Aalen estimator of the transition rates as

4 $$\begin{aligned} \widehat{{\varLambda }}_{jk}^{(\text {LM})}(t) := \int _{s}^{t} \frac{J_{j}^{(\text {LM})}(u) \mathrm {d}\overline{N}_{jk}^{(\text {LM})}(u)}{\overline{Y}_{j}^{(\text {LM})}(u)}, \end{aligned}$$

and let $\widehat{{\varvec{{\Lambda }}}}^{(\text {LM})}(t)$ be the matrix with (*j*, *k*)th element $\widehat{{\varLambda }}_{jk}^{(\text {LM})}(t)$ and diagonal element $\widehat{{\varLambda }}_{jj}^{(\text {LM})}(t) = - \sum _{k \not = j} \widehat{{\varLambda }}_{jk}^{(\text {LM})}(t)$. Then the landmark Aalen-Johansen (LMAJ) estimator of (1) presented by Putter and Spitoni (2018) is given by

$$\begin{aligned} \widehat{\mathbf {P}}_{l}^{\text {LMAJ}}(s,t)&:= e_{l} \prod _{u \in (s,t]} \left( \mathbf {I} + \varDelta \widehat{{\varvec{{\Lambda }}}}^{(\text {LM})}(u) \right) . \end{aligned}$$

### The hybrid landmark Aalen-Johansen estimator

An undesirable feature of landmark subsampling is a reduction of the number of individuals at risk used for estimation. As defined, the subsample reduce the at risk set for all transitions, including Markov transitions if such exist. An easy way of improving the estimation is by plugging in Nelson-Aalen estimates based on the landmark sample in the Aalen-Johansen (AJ) estimator only for transitions where the difference between the landmark Nelson-Aalen estimate and the full sample Nelson-Aalen estimates differ substantially. This is the main idea behind what we will refer to as the hybrid Aalen-Johansen (HAJ) estimator. We are therefore in particular interested in methods for detecting and deciding when differences in the hazard estimates are large enough to prefer the landmark estimate. In Section 3.2 we describe how a simple two-sample testing procedure can be used to identify the set of such non-Markov transitions. If this is only a subset of the full set of transitions *E*, we refer to the multi-state processes as *partially non-Markov* and denote the set of non-Markov transitions as $A \subset E$. More formally partially non-Markov means that a subset of transitions satisfies $E[\mathrm {d}N_{jk} \vert \mathcal {F}_{t-}, Y_{j}(t) = 1] = E[\mathrm {d}N_{jk} \vert Y_{j}(t) = 1]$, where $\mathcal {F}$ is the natural filtration of *X*. The HAJ estimator is a plug-in estimator of a transition rate estimator where rates are estimated as:

5 $$\begin{aligned} \widehat{{\varLambda }}_{jk}^{(\text {H})}(t) := \left\{ \begin{array}{ll} \widehat{{\varLambda }}_{jk}(t), &{} jk \not \in A; \\ \widehat{{\varLambda }}_{jk}^{(\text {LM})}(t), &{} jk \in A. \end{array} \right. \end{aligned}$$

Define $\widehat{{\varvec{{\Lambda }}}}^{(\text {H})}(t)$ to be the matrix with (*j*, *k*)th element $\widehat{{\varLambda }}_{jk}^{(\text {H})}(t)$ and diagonal element $\widehat{{\varLambda }}_{jj}^{(\text {H})}(t) = - \sum _{k \not = j} \widehat{{\varLambda }}_{jk}^{(\text {H})}(t)$. Now, the HAJ estimator of (1) is

$$\begin{aligned} \mathbf {\widehat{P}}_{l}^{\text {HAJ}}(s,t)&:= e_l \prod _{u \in (s,t]} (\mathbf {I} + \varDelta \widehat{{\varvec{{\Lambda }}}}^{(\text {H})}(u)). \end{aligned}$$

Observe that for $A = \emptyset$ we get the classical AJ estimator, while for $A = E$ we get the LMAJ estimator. Suppose that only a subset of transitions satisfy the Markov property above, and we wish to form the HAJ estimator. This can be done by applying the standard AJ estimator to a particular subset. More specifically such a subset is obtained by removing individuals that are not in the landmark state at the landmark time point from the specific risk sets used for estimating intensities for the non-Markov transitions. Applying the AJ estimator to such a reduced dataset from the landmark time point and onward will produce the HAJ estimate. As already mentioned, the LMAJ estimator is expensive; meaning that data reduction reduces precision (increases variance). The HAJ estimator will guarantee equal or better precision (relative to the LMAJ estimator), at the possible expense of introducing bias. In Sect. 4 we will study how these opposing effects balance out.

## Justification of the HAJ estimator

Our main reason for introducing the Hybrid estimator is to improve on the bias - variance trade off when estimating transition probabilities compared to the use of AJ or the LMAJ estimator. Before we turn to the HAJ estimator, we consider the basic estimation problem of estimating transition probabilities and how it changes as we move from the Markov to the non Markov case and from the AJ estimator to the LMAJ estimator.

### Product limits and transition probabilities

Estimation of transition probabilities in multi-state models relies on a special relation between conditional probabilities and cumulative hazard rate functions. The relation is the multi-state version of the argument for the Kaplan-Meier estimator in classical time-to-event models. For Markov multi-state models the result is due to Gill and Johansen (1990) and says that if $\mathbf {P}(s, t)$ is the transition probability matrix of a Markov multi-state model and ${\varvec{\Lambda }}$ the cumulative hazard rate matrix, then we have

6 $$\begin{aligned} \mathbf {P}(s, t) = \lim \prod _{m = 1}^{M} \mathbf {P}(t_{m-1},t_{m}) = \lim \prod _{m = 1}^{M} \left( \mathbf {I} + {\varvec{\Lambda }}(t_{m}) - {\varvec{\Lambda }}(t_{m-1}) \right) , \end{aligned}$$

where the limits are taken over refinements of (*s*, *t*], and $s =t_0< t_1< \cdots <t_M=t$. As a consequence, the product integral, when considered as a functional7is a convenient construct for producing plug-in estimators of probabilities in multi-state models based on estimators of the cumulative hazard rate matrix. In the Markov case Duhamel’s equation (see e.g. Andersen et al. (1993, Chapter 2)) and smoothness properties of (7) can be used to establish consistency and large sample properties either through Martingale methods (see e.g. Andersen et al. (1993, p. 320)) or through the continuous mapping theorem and the functional delta method. These results rely almost entirely on the properties of (7). Hence if, in the non-Markov case, one can establish (6), or rather a suitably modified version of (6), then there is good reason to believe that consistency and large sample behavior follow as well. We shall first argue heuristically that such a suitable modification of (6) exists in the non-Markov case and as in the Markov case there are two ways of deriving this result. In the Markov case, Gill and Johansen (1990) derived (6) directly by considering limit behaviour of the two products considered in (6). An alternative proof of (6) in the Markov case is due to Aalen et al. (2001), and alternatively by Andersen et al. (1993, p. 296), using what we shall refer to as a book keeping argument.

We shall first consider the direct approach and here we will rely entirely on the results of Overgaard (2019) (who derives the results for state occupation probabilities) and simply claim that the same arguments carry over. The one line argument is that “transition probabilities are occupation probabilities when we stratify to the conditioning event” (i.e. for the landmark data). Consider a fixed landmark time point *s* and landmark state *l* and define $\mathbf {P} ^{\text {LM}}(t, u) = \Bigl ( P_{jk}^{\text {LM}}(t, u) \Bigr )$ to be the matrix of transition probabilities in *the landmark population*, with

$$\begin{aligned} P_{jk}^{\text {LM}}(t, u) := \mathsf {P}(X(u) = k \, | \,X(t) = j, X(s) = l). \end{aligned}$$

By construction, and regardless of the Markov assumption, we have for $s \le t$

8 $$\begin{aligned} \mathbf {P}_{l}(s,t) = e_l \mathbf {P}^{\text {LM}}(t_{0},t_{1}) \cdots \mathbf {P}^{\text {LM}}(t_{M-1},t_{M}) , \end{aligned}$$

for $s = t_{0}< t_{1}< \cdots < t_{M} = t$. For a sufficiently fine partition of the interval (*s*, *t*] consider the approximation $\mathbf {P}^{\text {LM}}(t_{l-1}, t_{l}) \approx I + {\varvec{\Lambda }}^{(\text {LM})}(t_{l}) - {\varvec{\Lambda }}^{(\text {LM})}(t_{l-1})$ where ${\varLambda }_{jk}^{(\text {LM})}(\mathrm {d}t)$ is the transition rate of the landmark population, i.e.

$$\begin{aligned} {\varLambda }_{jk}^{(\text {LM})}(\mathrm {d}t) = \mathsf {E}\left[ \mathrm {d} N_{jk}^{(\text {LM})}(t) \, | \,Y_{1j}^{(\text {LM})}(t) = 1 \right] . \end{aligned}$$

The desired result is then to achieve the equality

9 $$\begin{aligned} \lim \prod _{m = 1}^{M} \mathbf {P}^{\text {LM}}(t_{m-1},t_{m}) = \lim \prod _{m = 1}^{M} \left( \mathbf {I} + {\varvec{\Lambda }}^{(\text {LM})}(t_{m}) - {\varvec{\Lambda }}^{(\text {LM})}(t_{m-1}) \right) , \end{aligned}$$

where the limits are taken over refinements of (*s*, *t*]. A detailed argument for this will take us too far astray and we will instead refer to Overgaard (2019) Theorem 2 and Theorem 5. Now we turn to the book keeping argument.

Assuming no censoring one can, as pointed out by Aalen et al. (2001) and alternatively by Andersen et al. (1993, p. 296), derive the landmark estimator of $\mathbf {P}_{l}(s,t)$ pointwise for (s,t) from a bookkeeping argument, which establishes an empirical version of (8). In order to do so let us briefly define

$$\begin{aligned} \widehat{\mathbf {P}}^{(\text {LM})}(s,t) := \prod _{u \in (s, t]}(\mathbf {I} + \varDelta \widehat{{\varvec{{\Lambda }}}}^{(\text {LM})}(u)). \end{aligned}$$

A natural way of estimating the transition probabilities $\mathbf {P}_{l}(s,t)$ for specific time points *s* and *t* is to consider the fraction of empirical means $\mathbf {\overline{Y}}^{(\text {LM})}(t) / \overline{Y}_{\bullet }^{(\text {LM})}(s)$. The number of individuals from the landmark sample in state *j* just after time *s*, $\overline{Y}_{j}^{(\text {LM})}(s + \varDelta t)$, may be expressed as those who were in state j at time *s* plus the net arrivals in the small time frame $\varDelta t$. That is

$$\begin{aligned} \overline{Y}_{j}^{(\text {LM})}(s + \varDelta t)&= \overline{Y}_{j}^{(\text {LM})}(s) + \sum _{k \ne j} (\overline{N}_{kj}^{(\text {LM})}(s + \varDelta t) - \overline{N}_{kj}^{(\text {LM})}(s)) \\&\quad -\sum _{k \ne j} (\overline{N}_{jk}^{(\text {LM})}(s + \varDelta t) - \overline{N}_{jk}^{(\text {LM})}(s)) \\&\quad \rightarrow \mathbf {\overline{Y}}^{(\text {LM})}(s) [\mathbf {I} + \varDelta \widehat{{\varvec{{\Lambda }}}}^{(\text {LM})}(s +)]_{j} \end{aligned}$$

for $\varDelta t$ $\rightarrow 0$, where $[\mathbf {I} + \varDelta \widehat{{\varvec{{\Lambda }}}}^{(\text {LM})}(s +)]_{j}$ denotes the *j*th column of $I + \varDelta \widehat{{\varvec{{\Lambda }}}}^{(\text {LM})}(s +)$. From this observation one obtains the following algebraic relation

10 $$\begin{aligned} \frac{ \mathbf {\overline{Y}}^{(\text {LM})}(t) }{\overline{Y}_{\bullet }^{(\text {LM})}(s)}&= \frac{\mathbf {\overline{Y}}^{(\text {LM})}(s) \widehat{\mathbf {P}}^{(\text {LM})}(s,t) }{\overline{Y}_{\bullet }^{(\text {LM})}(s)} = e_{l} \widehat{\mathbf {P}}^{(\text {LM})}(s,t) . \end{aligned}$$

We may recognize the right hand side as a continuous functional of the Nelson-Aalen transition rate matrix based on the landmark data. Hence consistency will follow from convergence of the left hand side to the desired transition probability, consistency of the rate matrix estimator and the continuous mapping theorem. The only difficulty here is the consistency of the rate matrix. For that we refer to appendix A or alternatively Nießl et al. (2020).

### The HAJ estimator

If $X_{1}, X_{2}, \ldots$ are partially non-Markov, then there is potential gain in terms of power and variance when using the HAJ estimator. Note that

11 $$\begin{aligned} \mathsf {E}\left[ \mathrm {d} N_{jk}^{(\text {LM})}(t) \, | \,Y_{j}^{(\text {LM})}(t) = 1 \right] = \mathsf {E}\left[ \mathrm {d} N_{jk}(t) \, | \,Y_{j}(t) = 1, X(s) = l \right] , \end{aligned}$$

which for Markov transitions $(jk) \in A$ implies that $\mathrm {d}{\varLambda }_{jk}^{(\text {LM})}(t) = \mathrm {d}{\varLambda }_{jk}(t)$. Likewise if we let $u \rightarrow {\varvec{\Lambda }}^{(\text {H})}(u)$ be the hybrid cumulative hazard rate matrix based on ${\varLambda }_{jk}$ for $(jk) \in A$ and ${\varLambda }_{jk}^{(\text {LM})}$, for $(jk) \in A^{C}$, we have ${\varvec{\Lambda }}^{(\text {LM})} = {\varvec{\Lambda }}^{(\text {H})}$. Thus, when (9) holds, the same holds in the partially non-Markov setting using ${\varvec{\Lambda }}^{(\text {H})}$ and consistency of the HAJ estimator follows by continuity of (7).

As pointed out by Titman and Putter (2020), from (11) it is clear that a test of the Markov assumption for a specific jump transition process from state *j* to state *k* can be obtained by comparing intensities based on disjoint landmark states. One can use a two-sample test of the hypothesis

$$\begin{aligned}&H_{0}:\mathsf {E}\left[ \mathrm {d} N_{jk}(t) \, | \,Y_{j}(t) = 1, X(s) = l_1 \right] \\&\quad =\mathsf {E}\left[ \mathrm {d} N_{jk}(t) \, | \,Y_{j}(t) = 1, X(s) = l_2 \right] \text { on } (s, \tau ]. \end{aligned}$$

Note that the two disjoint landmark states ensure independence between samples in the estimation procedure. Typically, $l_1$ would be the landmark state of main interest and $l_2$ the set of all other remaining possible states. In the application and the simulation study we use two different tests as selection criteria for determining Markov and non-Markov behaviour for specific transitions. Denote the log-rank test statistic for transition $j \rightarrow k$ from landmark time point *s* by $\mathfrak {X}_{s}$. This test statistic is the basis for a test referred to as the **point test**. Since the above test statistic is dependent on *s*, Titman and Putter (2020) suggest a more global test of the Markov assumption for transition $j \rightarrow k$ based on $\mathfrak {X} := \max _{i} \mathfrak {X}_{s_{i}}$, over a suitable grid $s_{1}, \ldots , s_{k}$. We refer to this as the **grid test**. For further discussion of the use of two sample tests to identify non-Markov transition see Online Resource C. We also stress that these tests are considered primarily as diagnostic tools and we do not address issues related to multiple testing.

### Censoring, covariates and variance

For simplicity, we have so far not considered censoring. Censoring is not used in the simulation experiment and is not a major issue in the practical application. In time-to-event analysis censoring is generally the rule rather than the exception and a comment on the matter is appropriate. First of all, in the setting of multi-state models, stronger censoring assumptions are typically needed, compared to the regular survival setting (see e.g. Aalen et al. (2008, p. 123)). Overgaard (2019) derives the result (6), which would extend to (9), based on a form of independent censoring coined “the status independent observation assumption” and Glidden (2002) considers right censoring with strong independence assumptions requiring censoring to be independent of states. A similar independent censoring assumption is needed for the LMAJ and HAJ estimators, as discussed by Putter and Spitoni (2018). For dependent censoring, Datta and Satten (2002) consider an IPCW version of the AJ estimator of state occupation probabilities. This weighted estimator was empirically investigated by Gunnes et al. (2007) and showed reasonable results for non-Markov behaviour induced by a joint frailty at baseline and various dependent censoring regimes. If present, dependent censoring can affect selection into the landmark sample, and a similar IPCW version of LMAJ or HAJ should be considered.

We have not considered covariate based hazard rate models in our formal treatment of the HAJ estimator but the results are expected to generalize to classical covariate models e.g. Cox or additive hazard regression due to the smoothness property of (7). See e.g. Hoff et al. (2019) for a discussion of covariate based models for the transition rates in relation to the LMAJ estimator.

Regarding variance estimates for the LMAJ and HAJ estimator we recommend bootstrapping. The LMAJ and HAJ estimator are constructed by applying the AJ estimator to different subsets of the entire data set. Non-parametric bootstrap estimates of the variance and point wise 95 percentile intervals can be obtained in the following way: First perform random re-sampling of subjects with replacements, then obtain estimates of transition probabilities (AJ, LMAJ and HAJ) by applying the AJ estimator to the different subsets of each of the the bootstrap samples. Variance and percentile estimates can then be obtained by pointwise sample variance estimates and pointwise sample percentile estimates. Since we use a test statistic to create the Hybrid estimator a natural question is whether to base the test on the original sample or to apply it to each bootstrap sample. In the practical application the tests are based on the original sample. Many of the classical variance estimators for the AJ estimator rely on the transition rates not depending on the history of the multi-state process. This is true under the Markov assumption but if we relax the Markov assumption additional variation is introduced for the Nelson-Aalen estimator (See appendix A for a formal discussion) and in turn for the plug-in estimator of transition probabilities. In the first simulation experiment we study the empirical coverage of the Greenwood type estimator of confidence intervals and in the practical application we compare such confidence intervals to bootstrapped confidence intervals. The results are included in Online Resource A.2 and B.2. The results suggest only minor deviations of the Greenwood type estimator, but it is unclear whether this holds in general.

## A simulation study

A central feature of our motivating data application on sickness absence and work participation is recurrent periods of sick leave. Since previous individual health history is very likely to impact future events of sick leave, we expect to see non-Markov behaviour for various transitions in our model. Furthermore, we expect a considerable amount of individual heterogeneity in a number of transitions due to for example variations in socioeconomic status, educational and professional backgrounds. Motivated by these problems we will focus on a multi-state simulation experiment with recurrent events and transition intensities subject to frailty effects. The smallest relevant multi-state model for such an investigation is the illness-death model with recovery depicted in Fig. 1. However, the appropriate analogy to our application will not be illness and death. Rather we will think of state 1 as employed, state 2 as on sick leave and state 3 as permanent disability.

We consider two types of experiments using non-Markov models over the time interval $[0, \tau ]$, with $\tau = 1000$. In both experiments the intensities of the transition counting processes are given by

$$\begin{aligned} \lambda _{jk} = V_{jk} \alpha _{jk}, \quad \text {for } jk \in \{(1,2), (1,3), (2,1), (2,3)\}, \end{aligned}$$

where $(\alpha _{12}, \alpha _{13}, \alpha _{21}, \alpha _{23}) = (0.12, 0.03, 0.15, 0.1)$ and $V_{jk}$ are individual frailties. The simulation experiments were performed 1000 times and each experiment had a total sample size of 1000 individuals. As the model is given conditional on the frailty, an extra step is needed to obtain the “true”, unconditional transition probabilities. They were calculated from a separate simulation experiment as the mean over 1000 repetitions of the LMAJ estimator applied to each simulated experiment, each of which also with a sample size of 1000 individuals. Note however that the LMAJ estimates here are based on landmark subsamples which will have somewhat lower sample size than the total sample size of 1000. The asymptotic distribution of the point and grid tests is approximated from wild bootstrapping (see Titman and Putter (2020) for further detail) and based on 500 bootstrap samples using standardized compensated Poisson processes. The HAJ estimator is constructed using the grid test (see section 3.2) applied to all transitions. The landmark states used to create the two independent samples are state 1 and state 2. We hope to find that the HAJ estimator is a useful intermediary between the AJ and the LMAJ estimator. To investigate this claim we consider two experiments; one focused on how large frailty effects need to be for the HAJ estimator to be preferable to the AJ estimator and one focused on when the non-Markov behaviour is significant enough for the HAJ estimator to compete with the LMAJ estimator. The two experiments are:

1. $V_{12} = V_{13} = V_{23} = 1$ and $V_{21}$ is gamma distributed with mean 1 and variance $\sigma ^{2} \in \{0, 0.4, 1.2, 2\}$, ranging from no frailty (i.e. Markov) to heavily right-skewed frailty;
2. $V = (V_{1}, V_{2}, V_{3}, V_{4})$ is log-normal distributed with mean 1 and covariance matrix
  $$\begin{aligned} \varSigma \approx \begin{pmatrix} 0.80 &{} 0.57 &{} -0.35 &{} 0.37 \\ 0.57 &{} 0.42 &{} -0.12 &{} 0.19 \\ -0.35 &{} -0.12 &{} 0.96 &{} -0.63 \\ 0.37 &{} 0.19 &{} -0.63 &{} 0.45 \end{pmatrix}. \end{aligned}$$
  Then, $W = \log (V)$ is normally distributed with $EW_{j} = -\varSigma _{jj}/2$ and ${{\,\mathrm{cov}\,}}(W_{j}, W_{k}) = \log (1 +\varSigma _{jk})$.

As mentioned, the HAJ estimator can be seen as a compromise between two extremes and the two experiments investigate to what extend such a compromise is useful. In other words, do we need the HAJ estimator at all and if so how much do we gain by using it?

**Fig. 2:**
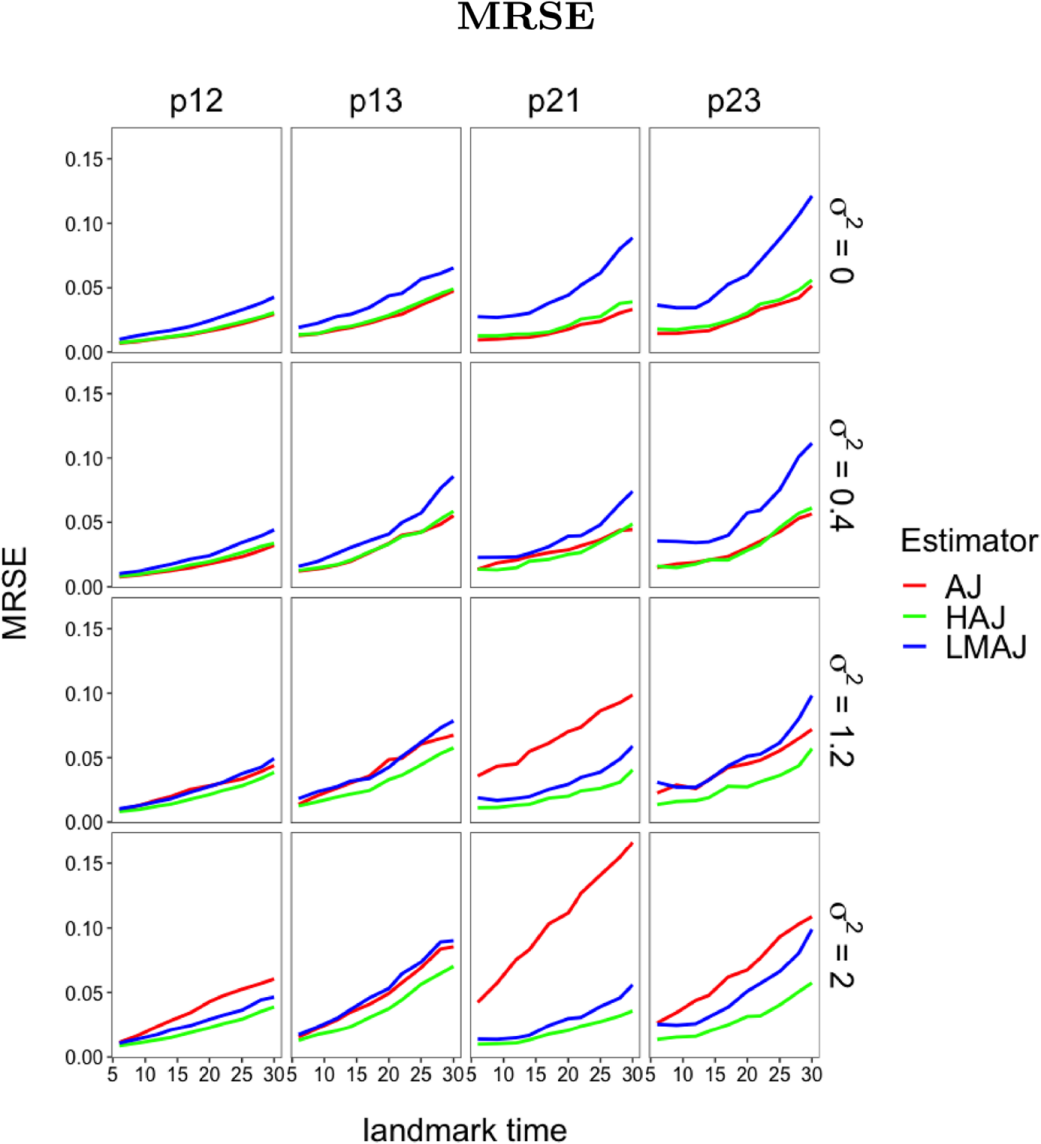
Experiment one: Mean residual squared error for estimators (AJ, HAJ and LMAJ) of the transition probabilities $p_{jk}$ from state *j* to state *k* as a function of landmark time points and frailty variance $\sigma ^{2}$. All numbers are based on 1000 samples where each sample has a size of 1000 individuals. The landmark grid is $\{6, 9, 12, 14, 17, 20, 22, 25, 28, 30\}$

**Fig. 3:**
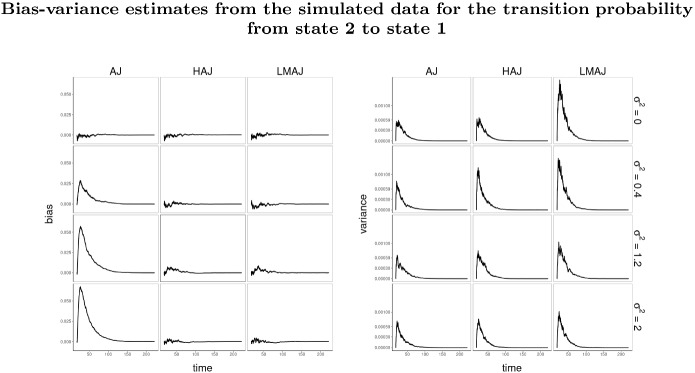
Bias and variance estimates of estimators of transition probability from state 2 to state 1 based on the AJ, HAJ and LMAJ estimator respectively. All estimates are computed from landmark time $s = 17$ and different levels of frailty variance $\sigma ^{2}$. Mean bias and variance estimates are based on 1000 samples, where each sample has a size of 1000 individuals

**Fig. 4:**
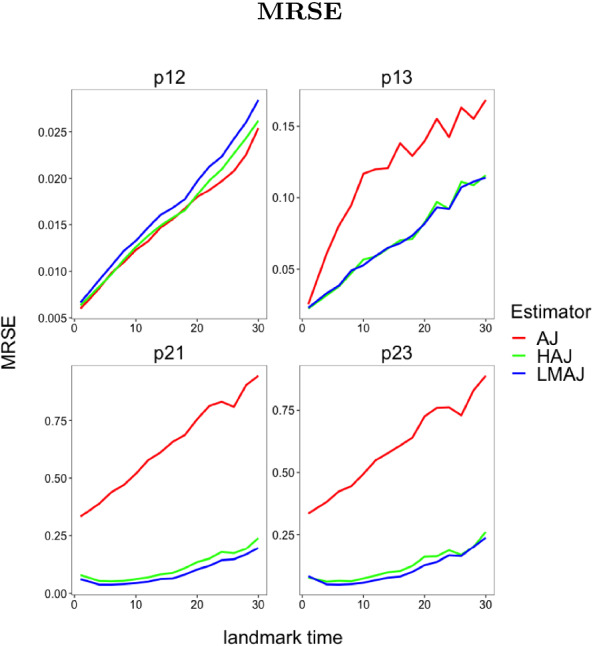
Experiment 2 (multivariate frailty): Mean residual squared error for estimators (AJ, HAJ and LMAJ) of the transition probabilities $p_{jk}$ from state *j* to state *k*. The landmark grid is $\{1, 4, 6, 8, 10, 12, 14, 16, 18, 20, 22, 24, 26, 28, 30\}$.

In the first experiment, all transitions are Markov unless the frailty variance $\sigma ^{2} > 0$, in which case we get a single non-Markov transition. The question is how large the frailty variance should be in order to detect a change in the transition probabilities. In particular we are interested in at what point the HAJ estimator starts to outperform the standard AJ estimator. In the second experiment all transitions are non-Markov and we investigate how the HAJ estimator performs compared to the LMAJ estimator under clear non-Markovian conditions induced by large correlated frailties (hence the choice of $\varSigma$). The approximation sign in the specification of $\varSigma$ is simply because $\varSigma$ is chosen numerically to ensure that the correlation matrix of *W* is positive definite. We evaluate the performance of the estimators using two performance measures. We consider pointwise (in time) empirical bias variance estimates (see Fig. 3) and mean residual squared error (MRSE) (see Figs. 2 and 4). MRSE is measured by the $L_{2}$ distance $\Vert f - g \Vert ^{2} = \int _{s}^{\tau } (f(t) - g(t))^2 \mathrm {d}t$ between estimates of $t \rightarrow P_{lk}(s,t)$ and the (simulated) true transition probability, with $\tau = 1000$. Here *s* is landmark grid times and the $L_{2}$-distance is calculated as a Riemann sum over all jump times. All empirical performance measures are produced based on 1000 simulations of each of the model specifications above. In both experiments the HAJ estimator is constructed using the grid test with a 5 percent significance level.

### Experiment 1

From Fig. 2 we see that, for non-zero frailty variance, the HAJ estimator performs at least as good as or better than the AJ estimator and better than the LMAJ estimator. In other words, the interpretation of the HAJ estimator as an intermediary between the AJ estimator and the LMAJ estimator seems to hold. In the Markov case, i.e. $\sigma ^{2} = 0$, we see almost no performance difference between the HAJ and the AJ estimators. Due to the in-built error from testing the Markov assumption we generally expect better performance of the AJ estimator over the HAJ estimator for Markov models, in particular for models with many transitions. For $\sigma ^{2} = 0.4$, the LMAJ estimator performs worse than the AJ estimator. The HAJ estimator is comparable to AJ for $\sigma ^2 = 0.4$ but starts to outperform AJ for larger values of $\sigma ^2$. In this experiment, HAJ always performed better than LMAJ. In other words, Fig. 2 suggests that if a perturbation of the transition intensity is sufficiently large, so as to induce significant non-Markov behaviour for the transition probabilities, then the HAJ estimator outperforms the AJ and the LMAJ estimator. However, as we shall see in the next experiment, this conclusion has its limitations. In the experiment all transitions are tested and results from the point tests and grid tests are included in Online Resource A.1.1.

Regarding the bias-variance trade-off, we see from Fig. 3 that the AJ estimator overestimates the transition probability $P_{21}(s, t)$, whereas the LMAJ and the HAJ estimator are close to the true transition probability. We also see that the HAJ estimator has smaller variance than the LMAJ estimator. This is exactly what we would expect from the HAJ estimator. Through the selection method (the grid test applied to all testable transitions) it accounts for the partly non-Markov and partly Markov behaviour, resulting in smaller bias than the AJ estimator and less variance than the LMAJ estimator.

### Experiment 2

From Fig. 4 we see that for the estimated transition probability from state 2 to 1 and state 2 to 3 the HAJ estimator performs slightly worse than the LMAJ estimator. This is expected when the non-Markov behaviour is strong enough, simply because the selection procedure for the HAJ estimator will make the wrong choice in some percentage of cases based on the significance level. The point at which the HAJ estimator is favourable to either the LMAJ or the AJ estimator depends partly on sample size and partly on how attenuated the non-Markov behaviour is. A good selection mechanism should take both elements into account. If we use a test as selection mechanism one could regard the significance level as a parameter deciding what “too much attenuation” should mean. From this perspective a standard 5 percent level need not be the optimal choice. In the experiment all transitions are tested and results from the point tests and grid tests are included in Online Resource A.1.2.

## An application to Norwegian registry data on sick leave, disability and work participation

**Fig. 5:**
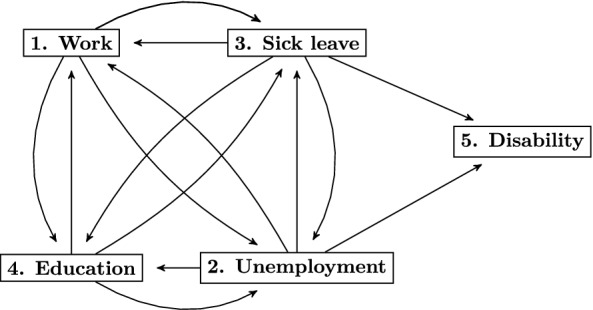
A multi-state model for work, education and health-related absence from work

To illustrate the HAJ estimator and compare it to LMAJ and AJ estimator, we consider a multi-state model with five states related to work participation. The proposed model is shown in Fig. 5 and consists of the following states: (1) work, (2) unemployment, (3) sick leave, (4) education (above high school) and (5) disability, where disability is an absorbing state. sick leave is defined here as paid partial or full long-term sick leave (16 $\ge$ calendar days). Individual multi-state histories through these states are constructed using data from various Norwegian national registries with data on employment, education and welfare benefits. For more details on the data material and source registries, see Hoff et al. (2019). In the dataset, information is available for the period 1992–2011 for all Norwegian males born between 1971 and 1976 (n = 184 951). Additionally, several individual covariates, on socioeconomic background, health, acquired education levels and results from military conscript examination, were available. We included individuals in the study from the 1st of July the year they turned 21 (1992–1997) and observed them for 14.5 years, until 31st of December (2006–2011). The time scale used in the model is days since inclusion, so that transition times are aligned on age and season, but not year.

One should realise that there are several potential violations of the Markov assumption for the model represented by Fig. 5. For example, it is plausible that individuals who have been working for a longer period are in more stable positions, further prolonging their stay in employment. Another example is individuals on long-term sick leave due to serious diseases, who may have lower probabilities for returning to work than people with minor illnesses. There are also laws and regulations that limit the possible duration of stays in states that are based on welfare benefits. Based on these circumstances we consider two examples for the comparison of the AJ, LMAJ and HAJ estimators. In particular, we look at transitions from sick leave at time $t = 100$ (100 days since inclusion) and transitions from unemployment at time $t = 3000$ (3000 days since inclusion). Note that these time points are chosen rather arbitrarily, to represent an early and late phase of the follow-up period. In the two examples that follows, we have also for illustrative purposes reduced the original dataset from Hoff et al. (2019) by looking at specific strata of covariate values.

**Fig. 6:**
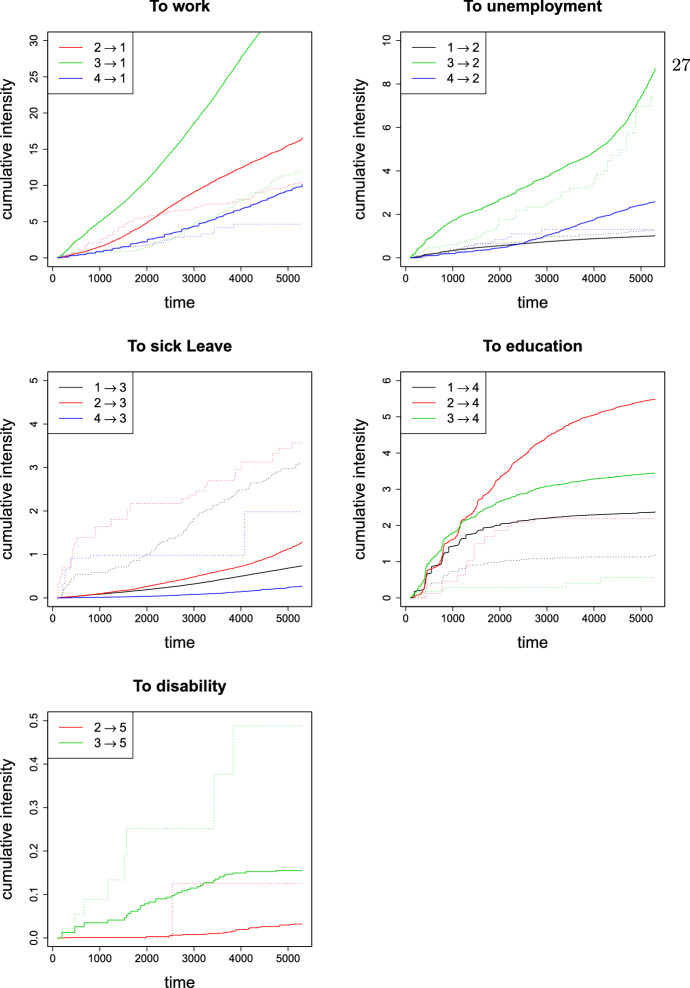
Cumulative transition intensities starting at landmark time-point $s = 100$ days. Full drawn lines are Nelson-Aalen estimates based on the reduced cohort ($n=23288$), while dotted lines are Nelson-Aalen estimates based on the landmark sample of individuals in sick leave ($n=72$)

### Example 1: Transitions from sick leave at $t = 100$

In our first example, we calculate transition probabilities from being on sick leave at day 100. The main motivation is to compare the AJ estimator with the LMAJ and HAJ estimator of transition probabilities for starting points after the original time of inclusion in the study (as this is where the AJ estimator may be biased due to violations of the Markov assumption). Note however, that, as in this example where time is measured as within year calendar time, the scientific question at t= 100 and t = 0 could indeed be slightly different. For example, using this time scale allows for investigating seasonal effects on the probability of returning to work from sick leave. In this particular analysis, the full dataset is reduced from 184 951 individuals to 23 288 by fixing a number of covariates. Specifically, we consider high school completers attending general education (non-vocational) that scored between 7 and 9 (i.e. high scores) on the cognitive test during military conscript examinations (scores range between 1-9 where 9 is considered the best). The landmark subset then consists of only 72 individuals. Due to the heavy restriction on covariates, we expect the sample population to now be fairly homogeneous compared to the full cohort. If the limited sample size makes the LMAJ low-powered for certain transitions, we would expect to see differences in the the LMAJ and HAJ estimates.

**Fig. 7:**
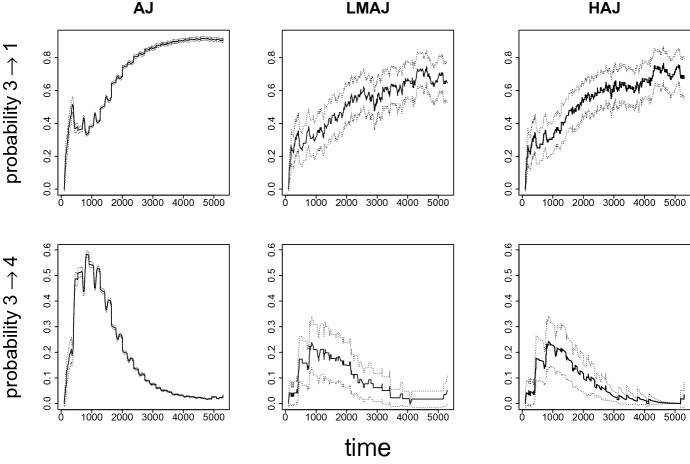
Estimated transition probabilities from sick leave to work ($3 \rightarrow 1$) and from sick leave to education ($3 \rightarrow 4$). Dotted lines are 95$\%$ bootstrap (1000 samples) confidence intervals. The landmark sample consists of 72 individuals on sick leave at day 100. The full sample includes 23288 individuals

We start by looking at cumulative transition intensities in the landmark sample and compare them with cumulative transition intensities calculated from the full dataset. The various intensities are shown in Fig. 6. Differences between the curves from the two data samples indicate a violation of the Markov assumption. Inspection of Fig. 6 suggests that transitions exhibiting similar intensities in the landmark sample and the full sample are $1 \rightarrow 2$, $4 \rightarrow 1$ and $4 \rightarrow 2$. In addition to visual inspection, we can test for non-Markov behaviour using the point test described in Sect. 3.2. Results from this test, found in Online Resource Table C.1, imply that transitions $1 \rightarrow 2$, $2 \rightarrow 5$, $4 \rightarrow 1$ and $4 \rightarrow 2$ are not significantly non-Markov. Based on the above results, the HAJ estimator will here utilize all available data for transitions ($1 \rightarrow 2$, $2 \rightarrow 5$, $4 \rightarrow 1$ and $4 \rightarrow 2$) and only the landmark data for the other transitions. We illustrate the resulting transition probabilities from the landmark state (sick leave) into work and into education in Fig. 7. Estimated transition probabilities to all states based on the HAJ and LMAJ estimators are found in Online Resource B.2.1.

In this example, LMAJ and HAJ estimates are in close agreement and differ substantially from the AJ estimates. The AJ transition probability estimates are far greater than estimates produced by the LMAJ and HAJ estimators, indicating that the AJ estimates are biased. Figure 7 show that for transition 3 $\rightarrow$ 1, the HAJ estimates appears slightly smoothed compared to LMAJ estimates, and for transition 3 $\rightarrow$ 4, the pointwise confidence intervals for the HAJ estimates are narrower at late times than the confidence intervals for the LMAJ estimates. The AJ estimates have more narrow confidence intervals due to the much larger sample size. Confidence intervals for the two landmark estimators are based on bootstrapping, while for AJ estimator, Greenwood plug-in estimates for standard errors were used. In Online Resource B.2.1, we also compare plug-in estimates of standard errors with bootstrap estimates for the HAJ estimator and find that they are very similar, with the bootstrap standard errors being slightly larger.

### Example 2: Transitions from unemployment at $t = 3000$

In our second example we still use data on high school completers of general education, but now consider individuals with cognitive scores between 4 and 6 (medium scores) and only individuals with parents who completed high school as their highest formal education. This amounts to a total of 10 451 individuals. Day 3000 is chosen as the landmark time point, and the landmark state is now unemployment. The landmark subset consists of 463 individuals. Results of log-rank tests for identifying Markov and non-Markov transitions can be found in Online Resource Table C.2. The results indicate that transitions $2 \rightarrow 5$, $3 \rightarrow 2$, $3 \rightarrow 4$, $3 \rightarrow 5$, $4 \rightarrow 1$ and $4 \rightarrow 3$ are Markov. Thus, all available data are used for these transitions when constructing the hybrid estimator.

**Fig. 8:**
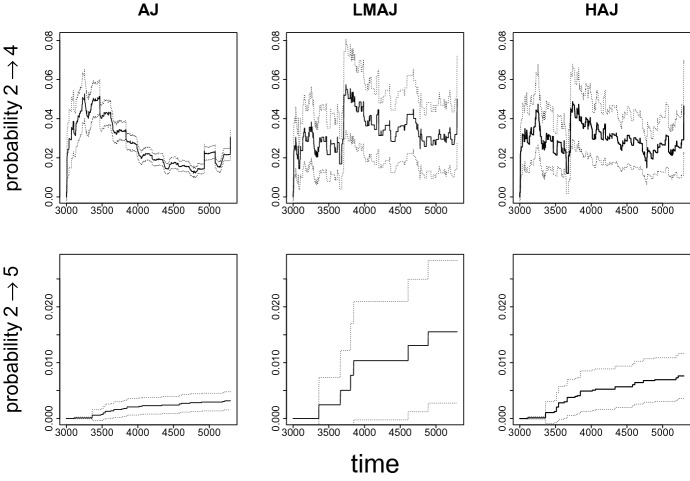
Estimated transition probabilities from unemployment to education ($2 \rightarrow 4$) and from unemployment to disability ($2 \rightarrow 5$). Dotted lines are 95$\%$ confidence intervals: model based for AJ and bootstrap (1000 samples) based for HAJ and LMAJ. The landmark sample consists of 463 individuals unemployed at day 3000. The full sample includes 10451 individuals

Estimates of transition probabilities from unemployment to education and disability are presented in Fig. 8. Compared to our previous example, AJ estimates seem less biased when compared to the estimates from the two landmark methods, but do fail to capture the development in the first one third of the time period. In terms of precision, the HAJ estimator seems to give higher precision than the LMAJ estimator for both the showcased transitions.

Estimated transition probabilities to all states based on the HAJ and LMAJ estimators and the corresponding comparison of bootstrap and Greenwood type estimates of standard errors for the HAJ estimator in this example can be found in Online Resource B.2.2.

## Discussion

The idea behind the HAJ estimator is to utilize the interpretation of transition probabilities as a functional of transition specific rates and provide a framework for analyzing how specific transitions affect the estimation of transition probabilities. This sensitivity analysis point of view is useful when modelling non-Markov multi-state data. First, it frames the problem of non-Markov behaviour as a more familiar problem of bias-variance trade-off by considering the HAJ estimator as a compromise between the AJ (low variance) and the LMAJ (low bias) estimators. As a rule of thumb, one would generally expect the HAJ estimator to have higher bias (since one allows for Markov behaviour to be assumed in specific transitions) and lower variance (due to the increased sample size) than the LMAJ estimator. The opposite, i.e. higher variance and lower bias, is generally expected when compared to the AJ estimator. Based on the simulation experiments it seems reasonable to believe that the lower variance comes at close to zero cost in bias. There are of course exceptions to this rule and one should be aware that, depending on the data generating mechanism, the HAJ estimator may in principle be superior (in terms of bias or variance) to both or neither of the alternatives (AJ and LMAJ). Secondly, with the HAJ estimator, one can think of the problem of non-Markov behaviour as a transition specific modelling choice, where certain transitions are more sensitive to non-Markov behaviour than others. Such considerations suggest a more comprehensive exploratory analysis of where, in a specific model, non-Markov behaviour can be problematic and where it might be negligible. If negligible, the simplicity and convenience of the Markov model is desirable and easier to report. In our construction of the HAJ estimator we focused on test statistics, but other tools such as judgement based on expert knowledge and visual inspection of plotted cumulative rates or transition probabilities can also be considered as selection mechanisms.

The application in Sect. 5 shows how the concept of partly non-Markov multi state is useful for real world data. The two examples in this section offer a more detailed investigation of long term work and sick leave trajectories for high school completers, following the analyses in Hoff et al. (2019). An even broader investigation would be possible using covariate adjusted rate models on the full data. However, the two examples illustrate the performance of the AJ, LMAJ and HAJ estimators in settings with medium to small transition specific risk sets. This will often be more representative for what you will see when using this type of registry data on sick leave and work participation to follow up smaller patient cohorts, such as in Gran et al. (2015), or when modelling other type of multi-state data, such as data describing clinical progress of hospital patients (Hazard et al. 2020).

The choice between the HAJ, AJ and LMAJ estimator depends on what kind of non-Markov behaviour one is dealing with and how pronounced it is in the data. Gunnes et al. (2007) investigated the Datta-Satten estimator of state occupation probabilities in non-Markov models and reached to some extent a similar conclusion; the benefit of using an estimator which can handle non-Markov behaviour and is prone to bias under Markov regimes depends heavily on how much and why the model in question deviates from the Markov property. In large cohort studies one often has to assume that heterogeneity will be a problem simply due to the complexity of the underlying data generating mechanisms. Even in cases where the non-Markov behaviour is negligible this seems like a problematic assumption to start from. Furthermore, a trivial but important advantage of the HAJ estimator over the LMAJ estimator is the increase in the sample size. Besides a reduction in variance this might in practice mean a difference between a feasible and an infeasible estimator. The HAJ estimator is therefore relevant for many applications of non-Markov and partially non-Markov multi-state models, in particular for studies of limited sample size, where the LMAJ estimator is not a viable option.

## Supplementary Information

The referenced Online Resources may be found online in the Supplementary Information section.

### Supplementary Information

Below is the link to the electronic supplementary material.

Supplementary material 1 (pdf 1396 KB)

## A On the estimation of the variance of transition probabilities for the LMAJ model

Glidden (2002, p. 366) states that the variance of the transition matrix is the same for Markov and non-Markov data. We shall argue that the variances are different, and that the one for the non-Markov case is not easy to estimate analytically. Our recommended approach, therefore, is bootstrapping.

Consider a number of independent individuals, $i=1,\ldots ,n$, and let $N_{jk}^{(i)}(t)$ be a counting process for transitions from *j* to *k* in the state space. The intensity process of individual *i* relative to the complete history of the individual up to time *t* is

$$\begin{aligned} \lambda _{jk}^{(i)}(t) = Y_{j}^{(i)}(t) Z_{jk}^{(i)}(t), \quad j,k \in E, \end{aligned}$$

where *E* is the state space and $Y_{j}^{(i)}(t)$ is 1 if the individual is in state *j* just prior to time *t* and zero otherwise. The process $Z_{jk}^{(i)}(t)$ is the (stochastic) transition rate from *j* to *k* for individual *i* given that it is in state *j* just prior to time *t* (meaning that the quantity $Y_{j}^{(i)}(t) Z_{jk}^{(i)}(t)$ is defined to be zero when the individual is not in state *j* just prior to time *t*). The *Z*-processes will generally differ between individuals according to the history of each individual. It is only when the process is Markovian that the transition rates will be fixed non-random quantities for all individuals.

According to counting process theory the process

$$\begin{aligned} M_{jk}^{(i)}(t) = N_{jk}^{(i)}(t) - \int _0^t \lambda _{jk}^{(i)}(s)ds \end{aligned}$$

is a martingale with respect to the history of individual *i* given certain regularity assumptions (see e.g. Aalen et al. 2008).

Since the individuals are independent, we can also define the history as the combined history of all individuals. The martingale property is then valid with respect to this combined history (due to independence). Define $N_{jk}(t)=\sum _i N_{jk}^{(i)}(t)$ and similarly for the other processes. Hence, summing over individuals we get the martingales

$$\begin{aligned} M_{jk}(t) = N_{jk}(t) - \int _0^t \lambda _{jk}(s)ds. \end{aligned}$$

Define

$$\begin{aligned} \overline{Z}_{jk}(t) = \frac{1}{Y_j(t)} \sum _i \left\{ Y_{j}^{(i)}(t) Z_{jk}^{(i)}(t) \right\} . \end{aligned}$$

Then

$$\begin{aligned} M_{jk}(t) = \int _0^t \left\{ dN_{jk}(s) - Y_j(s) \overline{Z}_{jk}(s)\right\} ds \end{aligned}$$

is a martingale. Hence, the following expression is also a martingale:

$$\begin{aligned} \overline{M}_{jk}(t) = \int _0^t J_j(s) \left\{ \frac{dN_{jk}(s)}{Y_j(s)} - \overline{Z}_{jk}(s)\right\} ds, \end{aligned}$$

where $J_j(s)=I(Y_j(s)>0)$ (Aalen et al. (2008, p. 88)).

The first part of the integral is the Nelson-Aalen estimator:

12 $$\begin{aligned} \widehat{{\varLambda }}_{jk}(t)= \int _0^t J_j(s) \frac{dN_{jk}(s)}{Y_j(s)} , \end{aligned}$$

and it follows that this is unbiased for the quantity ${\varLambda }_{jk}^{*}(t)=\int _0^t J_j(s) \overline{Z}_{jk}(s)ds$, and hence an asymptotically unbiased estimator for the cumulative mean rates ${\varLambda }_{jk}(t) := \int _0^t E(Z_{jk}(s))ds$. Under suitable regularity conditions we have

13 $$\begin{aligned} \widehat{{\varLambda }}_{jk}(t) - {\varLambda }_{jk}(t) = \overline{M}_{jk}(t) + {\varLambda }_{jk}^{*}(t) - \tilde{{\varLambda }}_{jk}(t) + o_{P}(1) \end{aligned}$$

for $\tilde{{\varLambda }}_{jk}(t) := \int _0^t J_j(s) E(Z_{jk}(s))ds$. From a standard martingale argument one may deduce the variance of the martingale (for $j \ne k$) $\overline{M}_{jk}(t)$ as

$$\begin{aligned} {[}\overline{M}_{jk}(t)]=\int _0^t J_j(s) \frac{dN_{jk}(s)}{Y_j(s)^2}. \end{aligned}$$

Furthermore, by independence of individuals and orthogonality (no common jumps) of the transition counting processes we get

$$\begin{aligned} {[}\overline{M}_{jj}(t), \overline{M}_{jk}(t)]&=-\int _0^t J_j(s) \frac{dN_{jk}(s)}{Y_j(s)^2} \text { for } j \ne k, \\ {[}\overline{M}_{jk}(t), \overline{M}_{j'k'}(t)]&= 0 \text { for } j \ne j' \text { and } \ne (k \ne k'), \\ {[}\overline{M}_{jj}(t)]&= \sum _{k \ne j}\ \int _0^t J_j(s) \frac{dN_{jk}(s)}{Y_j(s)^2}. \end{aligned}$$

The optional co-variation processes above are precisely the co-variation from the Nelson-Aalen estimator in the Markov case. However, from (13) we see that there is additional variation which is not accounted for in the estimates above. As a consequence, there will also be additional variation in either of the estimators: AJ, LMAJ and HAJ. However, it is unclear what the size of the bias is. The results in the Online Resource Section A.2 and B.2 seem to indicate that the bias of the standard Greenwood estimator is minor, but further studies of this are needed. It is generally not obvious how to estimate the additional variation from (13). Perhaps one could identify the additional variation under model specification of the hazard rates, but this is outside the scope of this article. In practice we suggest bootstrapping.

## Declarations

### Code availability

The R functions producing the simulation experiments (see sim_fun), the point test and the grid test (see wbMarkov) are part of the R-package multistate available on github: https://github.com/niklasmaltzahn/multistate. The package multistate has a number of additional convenience functions useful for landmarking and hybrid estimation. Furthermore, the data output of sim_fun is easily formatted for fitting multi-state models using the mstate package (de Wreede et al. 2011).
