# Supplementary material for: A hybrid landmark Aalen-Johansen estimator for transition probabilities in partially non-Markov multi-state models
Source: Lifetime Data Anal. 2021 Sep 30;27(4):737–60. doi: 10.1007/s10985-021-09534-4 (PMC8536588; doi:10.1007/s10985-021-09534-4)
Supplement: Supplementary file 1 — Supplementary material 1 (pdf 1396 KB) [file 10985_2021_9534_MOESM1_ESM.pdf]

Supplementary Information for *A hybrid landmark Aalen-Johansen estimator for transition probabilities in partially non-Markov multi-state models*

Niklas Maltzahn[1,2], Rune Hoff[1], Odd O. Aalen[2], Ingrid S. Mehlum[3], Hein Putter[4] and Jon Michael Gran[2,1]

September 27, 2021

Online Resource A

Supplementary material for the simulation study in Section 4 of the main document

A 1 Testing the Markov assumption

A 1.1 Experiment 1

From Figure A 1 we see that the larger the frailty variance described in Section 4 of the main document is (corresponding to a more right-skewed frailty distribution), the larger is the rejection rates of the point tests. We also see that the point tests for transitions without frailties are close to the 5 percent level. The shaded areas in the test plots are 95% pointwise Agresti-Coull confidence intervals (see e.g. Agresti and Coull ((1998))). These confidence intervals have shown to exhibit robust small sample properties and endpoint properties (i.e. with success rates close to zero or one). These are favourable features for the point tests since the sample size of the tests shrinks with increasingly late landmark time points. Furthermore, the true rejection rates of Markov transitions should be close to 0.05 (the α level) and endpoint robustness is therefore also desirable. The same type of confidence intervals are produced for the grid test. Some of the upper confidence limits of the grid test are above 1. This is due to the asymptotic formula for the intervals but of course practically impossible to achieve.

Corresponding author:Niklas Maltzahn, email:niklasmalt@gmail.com
[1]Oslo Centre for Biostatistics and Epidemiology, Oslo University Hospital, Norway
[2]Oslo Centre for Biostatistics and Epidemiology, Department of Biostatistics, University of Oslo, Norway
[3]National Institute of Occupational Health, Norway
[4]Leiden University Medical Center, Leiden University, The Netherlands

Rejection rates of point tests

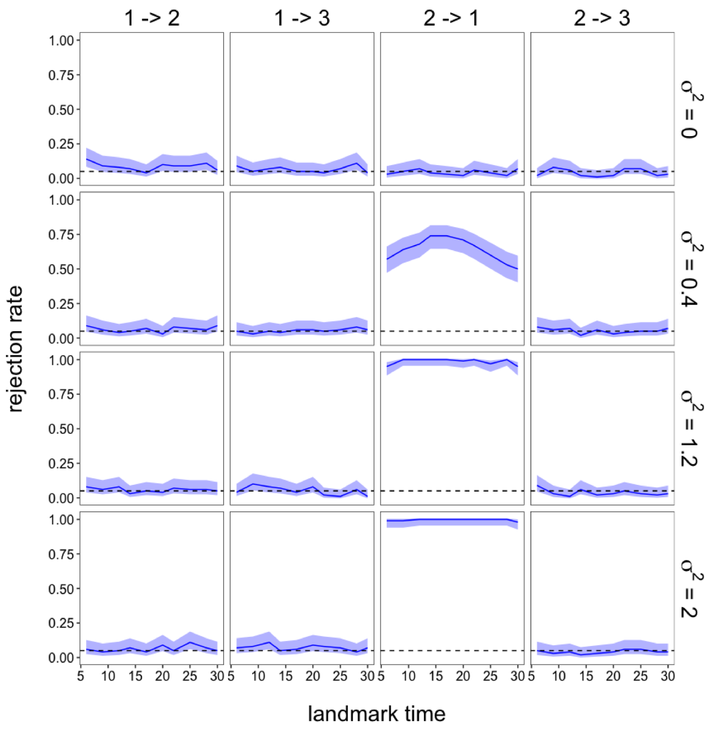

Fig. A 1: Experiment 1: Rejection rates of point tests with Agresti-Coull confidence intervals plotted against landmark time. All numbers are based on 1000 samples where each sample has a size of 1000 individuals. The landmark grid is $\{6, 9, 12, 14, 17, 20, 22, 25, 28, 30\}$

The grid tests in Table A 1 tell more or less the same story as the point tests in Figure A 1, but are slightly more conservative. This behaviour is expected because of the construction being a supremum of many point tests. Judging from the test statistics we see a clear indication of non-Markov behaviour in transition $2 \to 1$, but the main question is of course; is this behaviour sufficiently non-Markov to suggest a change of estimator? To answer this question we can consider the MRSE plots in the main document.

Grid-test rejection rates

Variance	Transitions	Mean	lower CI	upper CI
$\sigma^2 = 0.0$	$1 \to 2$	0.13	0.08	0.21
	$1 \to 3$	0.07	0.03	0.14
	$2 \to 1$	0.08	0.04	0.15
	$2 \to 3$	0.05	0.02	0.11
$\sigma^2 = 0.4$	$1 \to 2$	0.03	0.01	0.09
	$1 \to 3$	0.04	0.01	0.10
	$2 \to 1$	0.98	0.93	1.00
	$2 \to 3$	0.02	0.00	0.07
$\sigma^2 = 1.2$	$1 \to 2$	0.08	0.04	0.15
	$1 \to 3$	0.10	0.05	0.18
	$2 \to 1$	1.00	0.96	1.01
	$2 \to 3$	0.05	0.02	0.11
$\sigma^2 = 2.0$	$1 \to 2$	0.05	0.02	0.11
	$1 \to 3$	0.03	0.01	0.09
	$2 \to 1$	1.00	0.96	1.01
	$2 \to 3$	0.03	0.01	0.09

Table A 1: Experiment 1: Grid-test rejection rates for experiment 1, with upper and lower confidence bounds for each transition and each choice of frailty variance. All numbers are based on 1000 samples where each sample has a size of 1000 individuals. The landmark grid is $\{6, 9, 12, 14, 17, 20, 22, 25, 28, 30\}$

A 1.2 Experiment 2

When comparing the rejection rates of Figure A 2 (point test) or Table A 2 (grid test) with the MRSE plots for the transition probabilities in the main text, an interesting observation stands out. Looking at transitions $1 \to 2$ and $2 \to 3$, we see that differences in hazard estimates (suggested by the test statistics) for a particular transition, does not necessarily imply a difference between the corresponding transition probability estimates. One reason for this is that the transition probability really depends on the relation between multiple hazards rather than one particular hazard function and the net effect on the transition probability of heterogeneity in multiple transitions is not clear. Furthermore, from the model specification, the test procedure has some natural constraints in terms of which groups it is able to compare based on landmark time and state. In other words the groups we are able to compare based on landmarking are not necessarily those, which reveal the underlying heterogeneous effects. Therefore we can have situations where the test will not be able to detect significant differences although they exist.

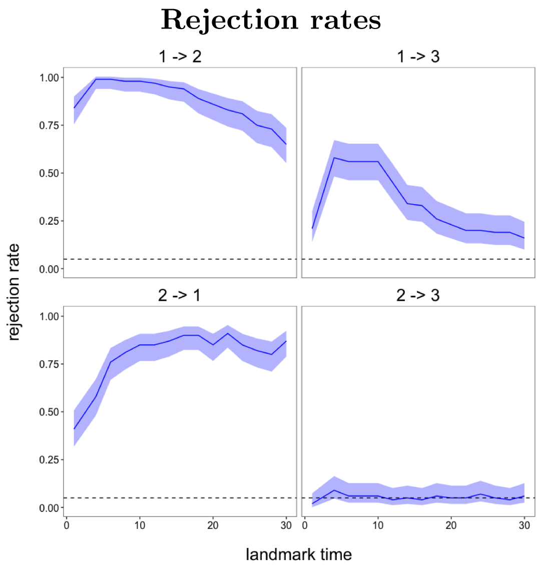

Fig. A 2: Experiment 2: Rejection rates for point tests with confidence intervals. The landmark grid is $\{1, 4, 6, 8, 10, 12, 14, 16, 18, 20, 22, 24, 26, 28, 30\}$.

	Mean	Lower CI	Upper CI
Transition $1 \to 2$	1.00	0.96	1.01
Transition $1 \to 3$	0.69	0.59	0.77
Transition $2 \to 1$	1.00	0.96	1.01
Transition $2 \to 3$	0.03	0.01	0.09

Table A 2: Experiment 2: Grid-test rejection rates for experiment 2 with upper and lower confidence bounds for each transition. All estimates are based on 1000 samples. Each sample with a size of 1000 individuals. The landmark grid is $\{1, 4, 6, 8, 10, 12, 14, 16, 18, 20, 22, 24, 26, 28, 30\}$.

A 2 Coverage and confidence intervals

To study the coverage and behaviour of Greenwood based confidence intervals we illustrate these for simulation experiment 1 and $\sigma^2 = 1.2$. See Figure A 3 and Figure A 4.

Empirical coverage of pointwise confidence intervals based on the Greenwood type estimates for $\sigma^2 = 1.2$

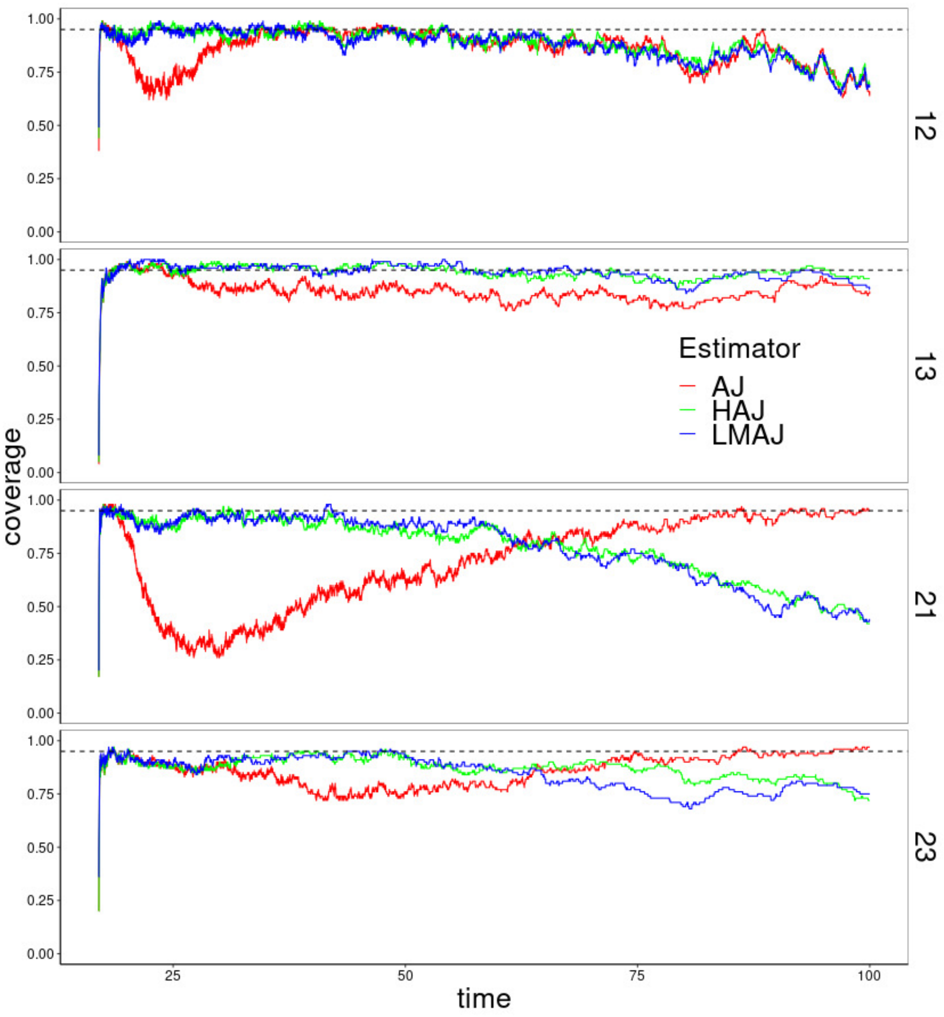

Fig. A 3: A plot of the empirical coverage of the Greenwood type estimator for the standard error for each of the Estimators: AJ, HAJ and LMAJ. Estimates are based on 100 simulations of model 1 with $\sigma^2 = 1.2$. All estimates are computed from landmark time $s = 17$. The true transition probability is the mean of LMAJ estimates based on 1000 simulations with 1000 individuals in each.

Confidence intervals for $\sigma^2 = 1.2$

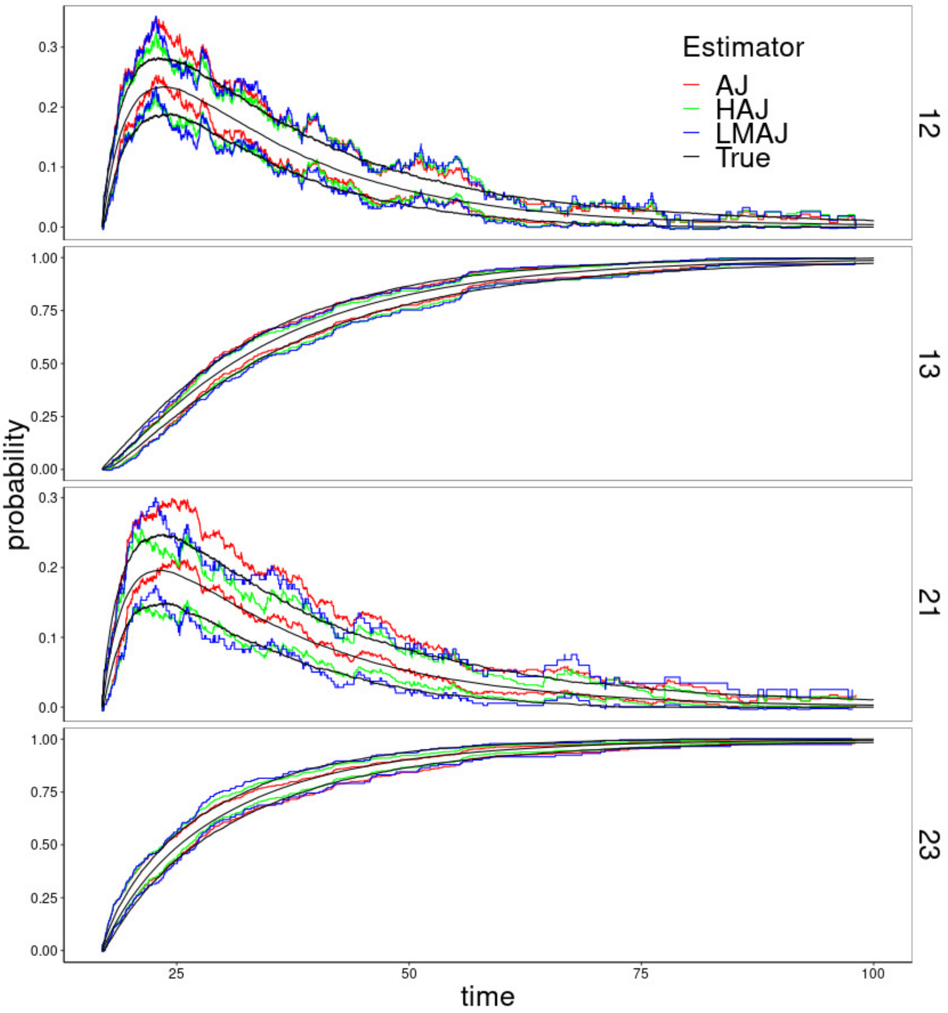

Fig. A 4: A plot of pointwise confidence intervals of transition probabilities based on the Greenwood type estimator of the standard error. We see estimates of AJ, HAJ and LMAJ based on one simulation of model 1 plotted against the (approximate) true pointwise percentile intervals (2.5 and 97.5 percentiles) for $\sigma^2 = 1.2$ and landmark time $s = 17$. The percentile intervals are given by the empirical 2.5 percentile and 97.5 percentile of LMAJ estimates based on 1000 simulations with 1000 individuals in each. The black line in the middle of the percentile intervals are the true transition probabilities, i.e, the pointwise empirical mean of LMAJ estimate based on the 1000 simulations.

Online Resource B

Supplementary material for the application in Section 5 of the main document

B 1 Testing the Markov assumption

A formal test for identifying Markov and non-Markov transitions can be based on the log-rank test as described in Section 3.2 of the main document, testing for differences in transition intensities in the landmark sample and the rest of the data (full dataset minus the landmark subset). Results for the two examples in Section 5 of the main article follows in Table B 1 and Table B 2. Contrary to the simulation experiment, the p-values are here based on standard log-rank asymptotics; i.e. based on the chi-square distribution and not on wild bootstrapping.

Transition	p-value	Transition	p-value
$2 \to 1$	0.000	$2 \to 3$	0.000
$3 \to 1$	0.000	$4 \to 3$	0.000
$4 \to 1$	0.067	$1 \to 4$	0.000
$1 \to 2$	0.206	$2 \to 4$	0.000
$3 \to 2$	0.000	$3 \to 4$	0.000
$4 \to 2$	0.255	$2 \to 5$	0.134
$1 \to 3$	0.000	$3 \to 5$	0.000

Table B 1: Results from log-rank tests of differences in transition intensities in the landmark subset and the rest of the data, testing the Markov assumption for Example 1 (Section 5.1). The landmark subset is made up by all individuals who were on sick leave at day 100.

Transition	p-value	Transition	p-value
$2 \to 1$	0.000	$2 \to 3$	0.000
$3 \to 1$	0.000	$4 \to 3$	0.835
$4 \to 1$	0.087	$1 \to 4$	0.000
$1 \to 2$	0.000	$2 \to 4$	0.037
$3 \to 2$	0.138	$3 \to 4$	0.343
$4 \to 2$	0.000	$2 \to 5$	0.196
$1 \to 3$	0.000	$3 \to 5$	0.108

Table B 2: Results from log-rank test on the difference in transition intensities in the landmark sample compared to the rest of the data, testing the Markov assumption for Example 2 (Section 5.2). The landmark sample is defined as all individuals in unemployment at day 3000.

B 2 Estimated transition probabilities and bootstrapped confidence intervals

To construct bootstrap confidence intervals for transition probabilities based on the HAJ estimator, we created 1000 bootstrap samples of the full dataset. From a full bootstrap sample we first create a bootstrap landmark sample. The bootstrap hybrid sample is then obtained by merging non-Markov transitions in the landmark sample with Markov transitions in the full bootstrap sample. The set of transitions deemed as Markov and non-Markov were assumed known in each bootstrap iteration, and set to that resulting from the formal test on the original non-bootstrap

dataset. For every bootstrap hybrid dataset, the transition probabilities were estimated and based on these estimates we calculated pointwise empirical standard errors.

B 2.1 Estimated transition probabilities for Example 1 (Section 5.1)

See Figure B 1 for estimated transition probabilities from state 3 (sick leave) and Figure B 2 for a comparison of the corresponding estimated bootstrap and Greenwood type standard errors.

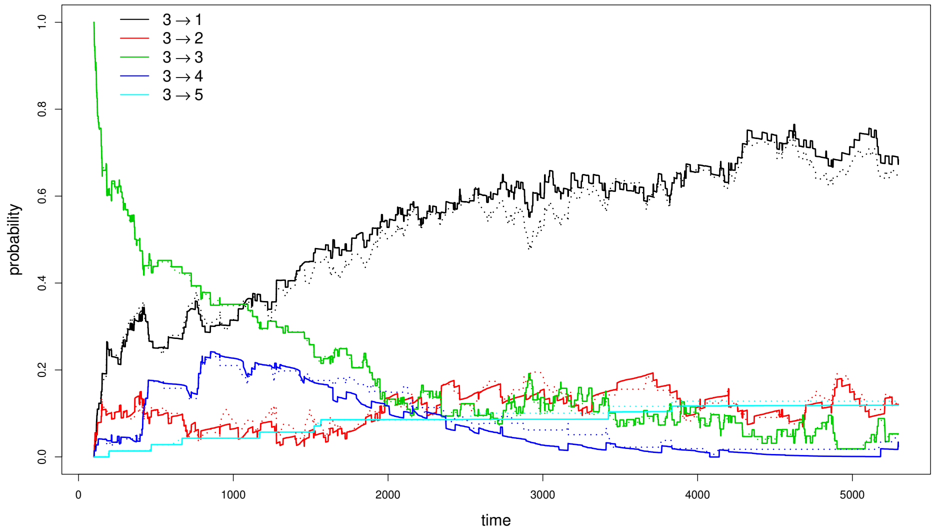

Fig. B 1: Estimated transition probabilities from in state 3 (sick leave) at time $t = 100$. Full drawn lines are estimates from the HAJ estimator, dotted lines are from the LMAJ estimator.

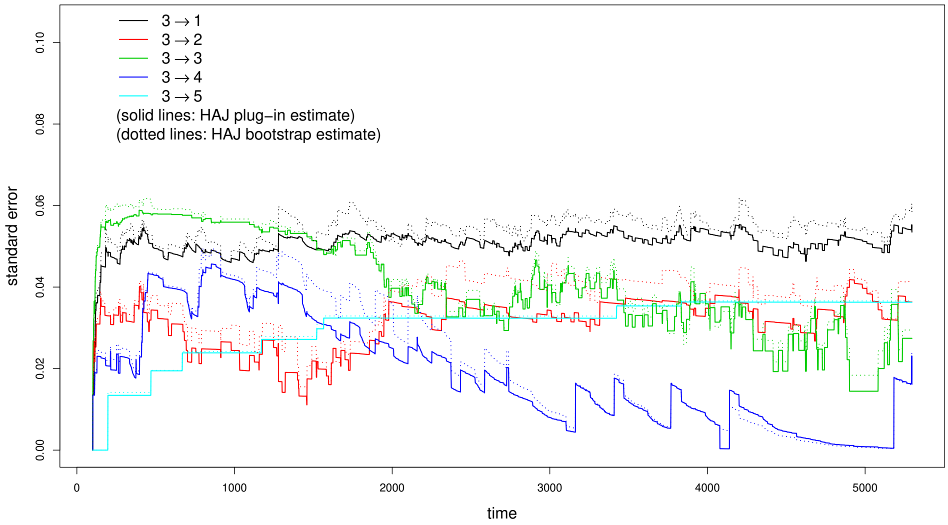

Fig. B 2: Bootstrap (using 1000 bootstrap samples) and Greenwood type standard errors for HAJ estimates of transition probabilities from state 3 (sick leave) at day 100.

B 2.2 Estimated transition probabilities for Example 2 (Section 5.2)

See Figure B 3 for estimated transition probabilities from state 2 (unemployment) and Figure B 4 for a comparison of the corresponding estimated bootstrap and Greenwood type standard errors.

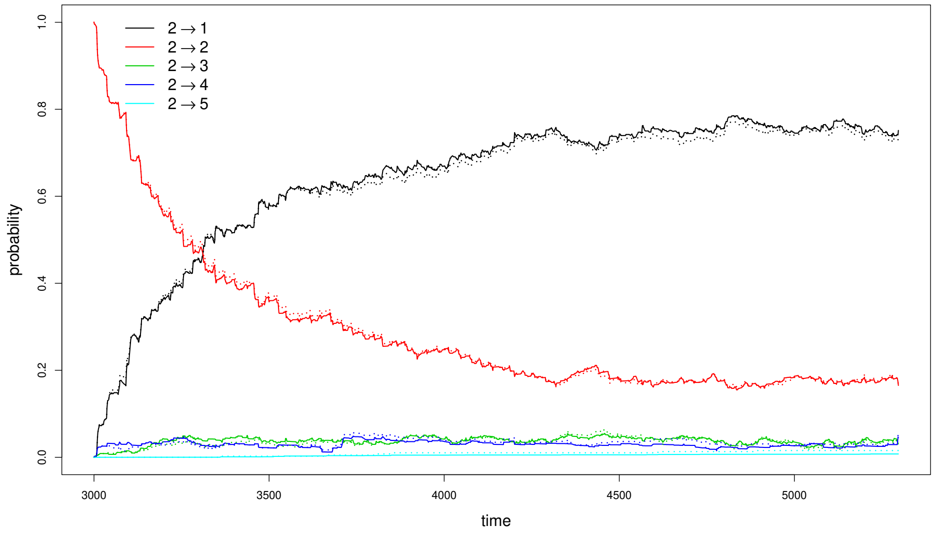

Fig. B 3: Estimated transition probabilities from in state 2 (unemployment) at day 3000. Full drawn lines are estimates from the HAJ estimator, dotted lines are from the LMAJ estimator.

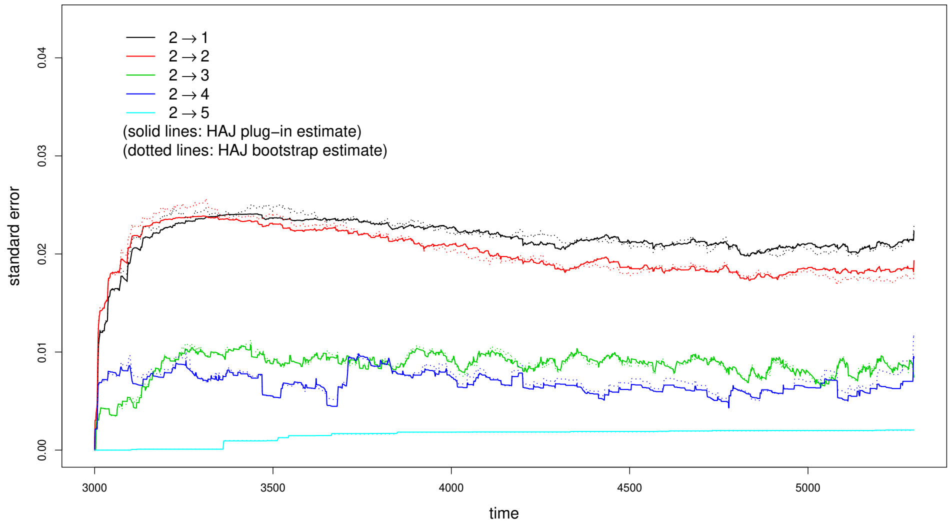

Fig. B 4: Bootstrap (using 1000 bootstrap samples) and Greenwood type standard errors for HAJ estimates of transition probabilities from state 2 (unemployment) at day 3000.

Online Resource C

On the identification of testable transitions

When constructing the HAJ estimator using two sample tests one needs to decide which transitions are amenable for testing. We will divide the complete set of transitions in the multi-state model into three disjoint sets $E = \mathcal{M} \cup \mathcal{N} \cup \mathcal{O}$. Here $\mathcal{M}$ is the set of Markov transitions (i.e., the set of transitions for which Markovianity is assumed without testing), $\mathcal{N}$ is the set of non-Markov transitions (for these landmark estimates are used in the HAJ), and $\mathcal{O}$ is the set of transitions that are open for testing. Obviously, different choices of $\mathcal{M}$, $\mathcal{N}$ and $\mathcal{O}$ may lead to different estimators. Choosing $\mathcal{N} = \mathcal{O} = \emptyset$ leads to the AJ estimator, while the choice of $\mathcal{M} = \mathcal{O} = \emptyset$ leads to the LMAJ estimator. An interesting observation is that many models will have untestable transitions. Take e.g. the transition from healthy state to death in an illness death model without recovery. In fact, if we by design cannot test a transition it is because the transition hazard estimate will be the same regardless of whether we landmark or not. Thus non-Markov behaviour only matters in so far as it is detectable in testable transitions. If we let $\mathcal{G} := (G, E)$ be the (bi)-directed graph representing transitions of X. A necessary and sufficient criteria for testing the transition $i \to j$ is the existence of subsets U, V in E satisfying:

$$\begin{aligned} &U \cap V = \emptyset \text{ and there exists paths } p_U, p_V \text{ with root notes in } U, \\ &\text{respectively } V \text{ such that } p_U \cap p_V = (i \to j). \end{aligned} \tag{C 1}$$

From the criteria (C 1) we also see that if there exists a loop from U to U, the transitions out of U can be tested, at least in principle.

References

A. Agresti and B. A. Coull. Approximate is better than “exact” for interval estimation of binomial proportions. *The American Statistician*, 52:119–126, 1998.
